# Area-level income inequality and oral health among Australian adults—A population-based multilevel study

**DOI:** 10.1371/journal.pone.0191438

**Published:** 2018-01-24

**Authors:** Ankur Singh, Jane Harford, José Leopoldo Ferreira Antunes, Marco A. Peres

**Affiliations:** 1 Australian Research Centre for Population Oral Health (ARCPOH), Adelaide Dental School, The University of Adelaide, Adelaide, Australia; 2 Departamento de Epidemiologia, Faculdade de Saúde Pública, Universidade de São Paulo, São Paulo, Brazil; University of Cyprus, CYPRUS

## Abstract

**Background:**

A lack of evidence exists on the association between area-level income inequality and oral health within Australia. This study examined associations between area-level income inequality and oral health outcomes (inadequate dentition (<21 teeth) and poor self-rated oral health) among Australian adults. Variations in the association between area-level income inequality and oral health outcomes according to area-level mean income were also assessed. Finally, household-income gradients in oral health outcomes according to area-level income inequality were compared.

**Methods:**

For the analyses, data on Australian dentate adults (n = 5,165 nested in 435 Local Government Areas (LGAs)) was obtained from the National Dental Telephone Interview Survey-2013. Multilevel multivariable logistic regression models with random intercept and fixed slopes were fitted to test associations between area-level income inequality and oral health outcomes, examine variations in associations according to area-level mean income, and examine variations in household-income gradients in outcomes according to area-level income inequality. Covariates included age, sex, LGA-level mean weekly household income, geographic remoteness and household income.

**Results:**

LGA-level income inequality was not associated with poor self-rated oral health and inversely associated with inadequate dentition (OR: 0.64; 95% CI: 0.48, 0.87) after adjusting for covariates. Inverse association between income inequality and inadequate dentition at the individual level was limited to LGAs within the highest tertile of mean weekly household income. Household income gradients in both outcomes showed poorer oral health at lower levels of household income. The household income gradients for inadequate dentition varied according to the LGA-level income inequality.

**Conclusion:**

Findings suggest that income inequality at the LGA-level in Australia is not positively associated with poorer oral health outcomes. Inverse association between income inequality and inadequate dentition is likely due to the contextual differences between Australia and other high-income countries.

## Background

Over 300 studies have investigated associations between area-level income inequality and outcomes of mortality and morbidity at global, national and sub-national levels [1]. Reviews on the hypothesized association between high income inequality and worse health outcomes have reported conflicting findings/conclusions [2–6]. Earlier reviews indicated that the association between high area-level income inequality and worse health outcomes is not universal, and limited to a few outcomes [2, 3]. However, recent reviews have found more support for a detrimental impact of area-level income inequality on health [5, 6]. A scoping review on area-level social inequality and oral health also reported that the collective evidence was suggestive of associations between high income inequality and worse oral health [7].

Oral diseases such as dental caries and periodontal disease and consequent loss of teeth are widely prevalent [8], associated with high economic costs, impact labor productivity [9], and negatively impact the quality of life [10, 11]. Inadequate dentition (having fewer than 21 teeth) is associated with poor quality of life [12, 13]. Self-rated oral health is a subjective marker of oral health linked to the general state of health and functional ability contributing independently to long-term well-being and satisfaction [14]. At a sub-national level, higher area-level income inequality has been associated with worse individual oral health outcomes in USA at state level [15], in Brazil at municipal level [16–18], and in Japan at the district level [19]. Variations in the presence of associations according to oral health outcomes are found [7]. No associations were reported for the outcomes of dental caries [20], periodontal disease [21], and lack of functional dentition [22].

Theoretical explanations (material, behavioural, psychosocial, structural and neo-material) are proposed to explain how high area-level income inequality leads to poor oral health outcomes. Leading explanations include psychosocial and neo-material theories [2–6]. According to the psychosocial theory, high levels of income inequality leads to poor oral health outcomes through depletion of psychosocial assets (social capital) and increase in psychosocial stressors (increased social evaluative threats) at the societal level. On the other hand, neo-material theorists postulate that the harmful effects of income inequality on health outcomes are due to the combined lack of material resources and healthy public policies at the societal level [7].

Increasing income inequalities within and between countries has become a global concern as a range of detrimental consequences on social and economic indicators including inequality of opportunity, negative impacts on economic growth and its sustainability, negative impacts on labour productivity, underinvestment in education, and political instability and conflict are well described [23–25]. Gini coefficients, derived from the Lorenz curve, measures the extent to which the distribution of income (or, in some cases, consumption expenditure) deviates from a perfectly equal distribution among individuals or households within an economy. Its value ranges from ranging from 0 (perfect equality) to 1 [26]. Income inequality within Australia has increased over the last three decades with an increase in the national estimates of Gini coefficients from 0.27 in 1982 to 0.32 in 2011–12 [27]. Compared to the general health literature from other high-income countries, limited and inconclusive evidence exists on the associations between area-level income inequality and health outcomes within Australia [28, 29]. No associations were reported between area-level income inequality and the outcomes of mental health at neighborhood and city level [29]. An ecological study reported positive associations between area-level income inequality and alcohol-related harms at the Local Government Area (LGA) level, while inverse associations were reported for the outcome of alcohol-attributable hospitalization [28].

Studies of the association between area-level income inequality and health are complicated by the known association between individual income and health [30] as well as that between area-level income and health [31]. Both may themselves be associated with the level of inequality. Thus, larger inequalities could result in poorer health overall because (at the same average income) it results in more people on low incomes–a compositional effect of inequalities [32] rather than, greater inequalities impacting on health at any level of individual income. These complications contribute to the ongoing debate about the pathways through which area-level income inequality may potentially affect health status [33] and to methodological developments, including multilevel analysis intended to account for potential confounding by both area and individual level factors [34].

Consequently, many studies both in general and oral health have applied the multilevel technique to investigate the associations between area-level income inequality and individual health outcomes as shown in literature reviews [6, 7]. A systematic review of multilevel studies on the associations between area-level income inequality and mortality, and self-rated health reported inverse association (odds ratio of 1.08) between high income inequality and poor self-rated health [5].

Recently, there has been a call to also investigate the impact of area-level income inequality on health inequalities within societies, not only average health [35]. To our knowledge, no study exists within Australia that examines associations between area-level income inequality and oral health outcomes. Additionally, none of the existing studies of general health outcome within Australia [28, 29] have applied a multilevel statistical analytical technique, despite its advantages, or explored the impact of area-level income inequalities on health inequalities.

Therefore, this study aimed to:

1. test associations between area-level income inequality and oral health outcomes of inadequate dentition and poor self-rated oral health at the individual level after accounting for both area and individual level confounders,
2. test the associations between area-level income inequality and oral health according to area-level mean income,
3. compare the associations between household income and the two oral health outcomes under different levels of area-level income inequality.

## Methods

### Study population

To address the objectives of the current study, a secondary analysis was conducted on the data available for dentate adults from National Dental Telephone Interview Survey (NDTIS) 2013. NDTIS is a nationwide cross-sectional population-based survey administered to monitor population levels of oral health across all states and territories conducted by the Australian Research Centre for Population Oral health (ARCPOH) every 2 ½ years since 1994. The survey involved a random sample of Australian residents aged five and over in all states and territories. An overlapping dual sampling frame design was adopted for the survey.

The first sampling frame was created from the electronic product ‘Australia on Disc 2012 Residential’ supplied by United Directory Systems. This product is an electronic listing of people/households listed in the White Pages telephone directory across Australia and is updated annually. Both landline and mobile telephone numbers were provided where applicable. A stratified two-stage sampling design was then adopted to select the sample from this sampling frame. Once a telephone contact was made with a selected household, one person aged ≥18 years was selected for the interview. A second sampling frame was used so as to include households that were not listed in the White Pages. This sampling frame was supplied by Sampleworx who supplied 20,000 mobile telephone numbers by appending randomly generated suffix numbers to all known Australian mobile prefix. More information on survey methodology is reported elsewhere [36].

Dentate adults aged ≥18 years (5,169 out of 6,340) within the survey were included in the analysis. This age-group was chosen for two reasons. First, provision of dental health services differs among individuals above and below the age of 18 years. Only children 17 years and below are eligible for Child Dental Benefits [37] in accordance with the Australian government policy. Second, a study reports that the magnitude of income inequality and health associations varies according to age groups, and the negative impact is predominantly observed among young adulthood [38].

Individual information for adults from NDTIS was allocated to multiple geographic levels through geocoding residential addresses obtained from the electronic white pages and self-reported questionnaire. For the purposes of analysis, LGAs were considered as an appropriate level of geography. LGAs represent the administrative boundaries for local government councils for the provision of a broad range of infrastructure, economic and community services to residents [39]. There are a total of 561 LGAs in Australia [39]. In the absence of information on residential addresses, individuals were allocated to LGAs using concordance files for postcodes to LGAs provided by the Australian Bureau of Statistics (ABS) [40].

### Data collection

Data was collected between May 2013 and March 2014 via telephone interview. Trained interviewers conducted telephone interviews using WinCATI® software. The collected data included measures of self-reported number of teeth and self-rated oral health status, use of and access to dental services, social impact of oral health, the financial burden of dental care, and private health insurance that covered dental expenses.

### Outcomes

Two outcomes were included in the study: inadequate dentition and self-rated oral health. Inadequate dentition was defined as having fewer than 21 teeth [12]. Individuals were asked ‘do you have any of your own natural teeth?’, and ‘there are 16 teeth, including wisdom teeth in the upper/lower jaw. How many teeth do you have remaining in your upper/lower jaw?’. Combining the responses to the two questions, a derived binary variable for each dentate individual was created to identify individuals with/without inadequate dentition. Adult proxy interviewees were not asked about the number of teeth, hence were excluded from this analysis. For the outcome of self-rated oral health, dentate participants were asked: ‘how would you rate your own dental health. Would you say that it is: excellent, very good, good, fair or poor.’ Responses of ‘fair’ and ‘poor’ were grouped as poor self-rated oral health, and ‘excellent’, ‘very good’ and ‘good’ were grouped together as better self-rated oral health.

### Exposure

The primary exposure was area-level income inequality measured by the Gini coefficient for LGAs with a range of 0 to 1. A value closer to 1 represents higher inequality compared to a value closer to 0. The values of Gini coefficients for each LGA were obtained from a published estimates [41] based on household incomes reported in the Australian Census of Population and Housing 2011. For this analysis LGAs were grouped into tertiles by their Gini coefficients (range: first tertile (0.292, 0.369); second tertile (0.370, 0.387); third tertile (0.388, 0.489)).

### Covariates

**Fig 1:**
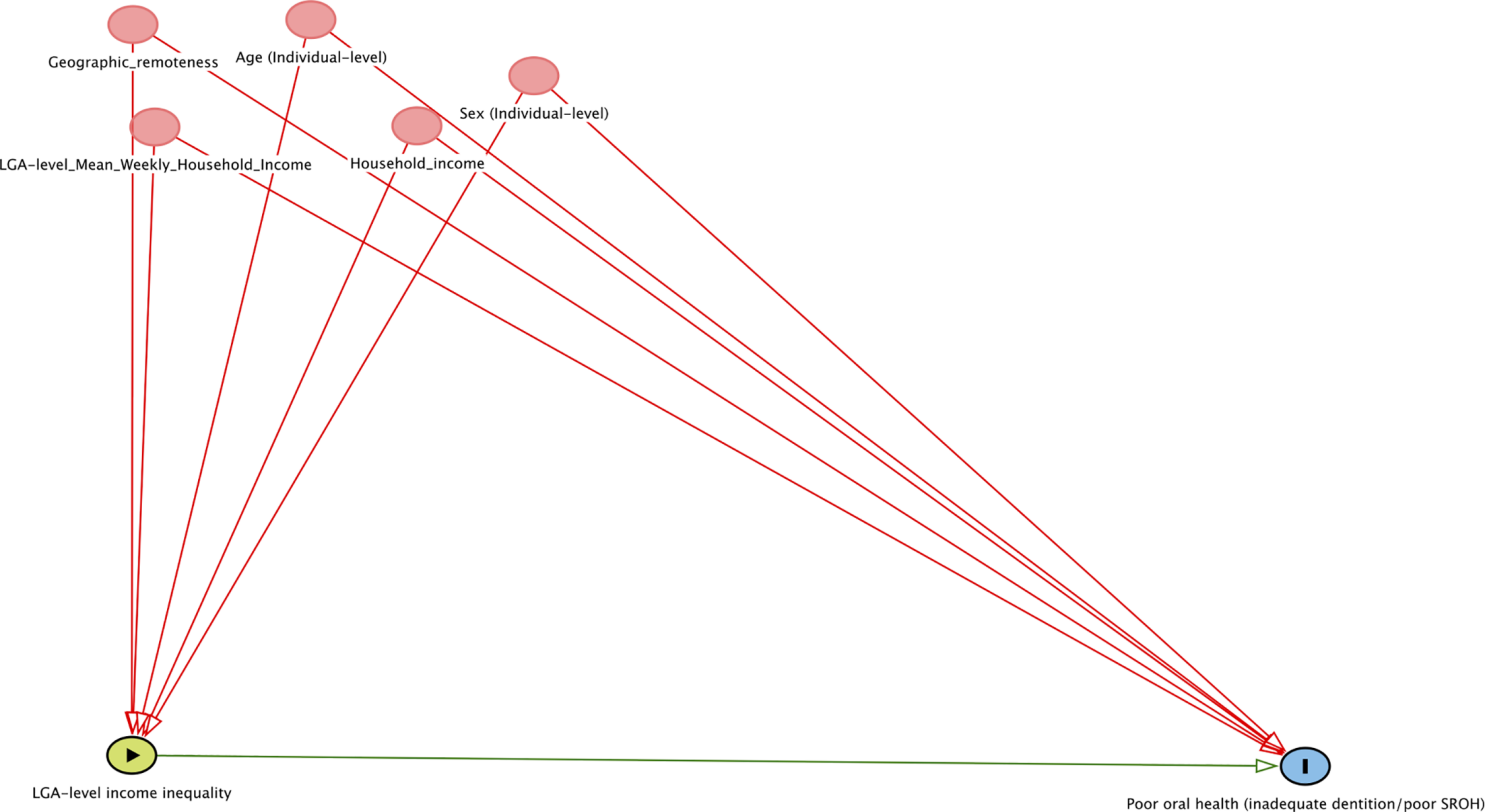
Directed Acyclic Graph (DAG) to represent the relationship between area-level income inequality and individual-level oral health outcomes.

Based on the evidence on the association between area-level income inequality and health [32], the individual-level and LGA-level covariates were included in the analysis. Age, sex and household income were included to address for confounding. Additionally, educational attainment was included to address for confounding specifically for aim 3. For LGAs, equivalised mean household income and geographic remoteness were included. The theorized relationship between LGA-level income inequality, oral health outcomes at the individual level, and covariates are shown through a Directed Acyclic Graph (DAG) (Fig 1). Age was treated as a continuous variable for analysis but categorized as 18 to 34, 35 to 54, 55 to 74 and, 75 years and above for descriptive purpose. Household income in Australian dollars was collected as a categorical variable and was further re-categorized in five groups. The new categories were: households having an annual income of less than $20,000, $20,000 to less than $50,000, $50,000 to less than $80,000, $80,000 to less than $100,000, and $100,000 and above. Geographic remoteness was recorded at the individual level and the categories included those residing in major city areas, inner regional areas, outer regional areas, and remote/very remote areas. LGA-level weekly mean equivalised household income was obtained from the Australian Census of Population and Housing 2011. Values were converted into tertiles for relative comparison between LGAs investigating potential social gradients in individual-level outcomes according to area-level income inequality. Tertiles were preferred over higher number of categories as the objective 2 of the study required examination of potential interactions between LGA-level income inequality and LGA-level weekly mean equivalised household income [42].

### Statistical analysis

The associations between LGA-level income inequality and individual oral health outcomes were modelled using multivariable multilevel logistic regression models with random intercepts and fixed slopes. Model 1 estimated the unadjusted association between the tertiles of Gini coefficients for LGAs and the two outcomes. Model 2 adjusted for age, sex, LGA-level weekly mean household income, household income and geographic remoteness. The direction and strength of association between LGA-level income inequality and the outcomes were estimated with a fixed parameter (odds ratio). Area-level heterogeneity in the outcomes and the variance explained by the inclusion of variables were estimated with random parameters (intra-class coefficient and median odds ratio) [43]. Stratified analyses of the association between LGA-level income inequality and the outcomes were conducted according to the tertiles of LGA-level weekly mean household income. The prevalence of both outcomes by household income was estimated from separate models for each of the high, medium and low tertiles of Gini. These models were adjusted for age, sex, educational attainment, LGA-level weekly mean household income and geographic remoteness. Survey commands (svy prefix) were used to account for the complex survey design and to perform the weighted descriptive analysis. All analyses were performed in Stata, v14. Five different sensitivity analyses were performed to confirm the robustness of the current findings. The rationale and method for each are presented in the supporting file (S2 Appendix).

## Results

Overall 6,340 adults were interviewed within NDTIS survey, with a participation rate of 34.3% (AIHW, 2016). A complete case analyses of 4,768 dentates nested in 428 LGAs for inadequate dentition, and 5,165 dentate adults nested in 435 LGAs for self-rated oral health were possible after excluding edentates (n = 307), and missing values for household income (n = 781), non-allocation to LGAs (n = 83), self-rated oral health (n = 4), and number of teeth (n = 401). Descriptive characteristics of the dentate adults from NDTIS 2013 is presented in Table 1. The sample had similar proportions of males and females and had relatively more individuals below the age of 54 years compared to those above. Comparisons between the characteristics of interviewed 5,978 dentates, full cases and demographic characteristics from Australian population census 2011 are presented in S1 Table. A flowchart is presented to explain the sample flow in S1 Fig.

**Table 1:** Descriptive characteristics of the sample according to the oral health outcomes (weighted percentages).

		Inadequate Dentition (n: 4,768; LGAs 428)		Self-Rated oral health (n: 5,165; LGAs: 435)	
Characteristics	Categories	% of sample	Inadequate dentition (%)	% of sample	Poor SROH (%)
Sex	Male	48.5	11.3	50.6	21.2
	Female	51.5	10.8	49.4	19.7
Age	18–34	28.7	0.4	30.1	14.6
	35–54	39.1	5.8	38.7	23.1
	55–74	26.0	21.9	25.1	23.2
	75 and above	6.1	47.7	6.1	21.5
Household Income	$100K and above	33.0	2.5	33.8	12.5
	80K < 100k	11.3	7.2	11.6	17.5
	50k < 80k	20.5	8.5	20.5	20.8
	20k < 50k	26.5	19.5	25.9	27.8
	Less than 20k	8.7	28.4	8.3	33.9
Educational attainment	Tertiary^a^	24.3	3.0	23.3	12.5
	Vocational^b^	47.6	12.6	47.3	22.7
	Student^c^	5.5	1.7	6.2	8.1
	Secondary^d^	22.7	18.6	23.2	27.3
Geographic remoteness	Major city	70.8	9.4	70.5	19.6
	Inner regional	18.7	15.4	18.8	21.1
	Outer regional	8.3	15.7	8.5	24.3
	Remote/Very remote	2.2	10.8	2.2	30.2
Inadequate dentition	No	89.0		89.0	18.5
	Yes	11.0		11.0	39.7
Self-rated oral health	Excellent/Very Good/Good	79.2	8.4	79.5	
	Poor/Very Poor	20.8	21.0	20.5	
Local Government Areas (LGAs)				Median	Range
Gini Coefficient 2011 household				0.377	0.292, 0.489
Mean weekly household income (2011) (Australian Dollars)				1577.6	823.6–3886.2
NDTIS Sample Size				7	1–446
		Categories	%	Gini Median	Gini Range
Gini Tertiles		Low	35.6	0.359	0.292, 0.369
		Medium	32.1	0.378	0.370, 0.387
		High	32.3	0.402	0.388, 0.489
Mean weekly household income2011 Australian Dollars (Range)		High (1750.2, 3886.2)	33.2	0.391	0.292, 0.472
		Medium (1420.8, 1748.6)	33.4	0.377	0.330–0.478
		Low (823.5, 1420.3)	33.4	0.370	0.312, 0.489

a: Bachelor/honors degree or more

b: Advanced diploma, diploma, associate degree, certificate level, and other qualifications

c: None completed but studying at university, TAFE apprentice, secondary school

d: No post-secondary qualification & not currently studying

The estimates obtained null models showed that the share of variance at the LGA level was higher for inadequate dentition (ICC: 4.3%, MOR: 1.44) than for the outcome of poor-self rated oral health (ICC: 1.05%, MOR: 1.20) (not reported in tables).

Unadjusted estimates obtained from model 1 showed that individuals in the most unequal LGAs had relative odds of 0.59 for inadequate dentition compared to individuals in the least unequal LGAs (Table 2). After adjusting for individual age, sex, household income, LGA mean household weekly income, and geographic remoteness, individuals in most unequal LGAs had relative odds of 0.64 of having inadequate dentition, with LGAs in the lowest tertile of Gini at reference. There were no differences between the low and middle inequality LGAs for inadequate dentition (Table 2). The median odds ratio (MOR) obtained from model 1 showed that median odds of inadequate dentition increased by 1.30 times with a move to an area with a higher probability of inadequate dentition. The inclusion of age, sex, household income and LGA-level mean income in model 2 reduced the MOR to 1.09 (Table 2). Results from the stratified analysis indicate that the lower odds of inadequate dentition in the highest tertile of income inequality were limited to LGAs with higher mean weekly household incomes (Table 2).

**Table 2:** Multilevel logistic regression analysis for the association between LGA level income inequality and inadequate dentition (No. of Areas = 428; N of individuals = 4,768).

		Model 1		Model 2	
	Categories	OR	95% CI	OR	95% CI
Income Inequality (Gini)	Low	1		1	
	Medium	1.10	0.89, 1.37	0.88	0.70, 1.11
	High	0.59	0.46, 0.75	0.64	0.48, 0.87
Mean weekly household income	High			1	
	Medium			1.44	1.12, 1.86
	Low			1.37	1.00, 1.88
Age	1-year change			1.07	1.06, 1.08
Sex	Male			1	
	Female			0.79	0.65, 0.96
Household Income	$100K and above			1	
	80K < 100k			1.79	1.13, 2.87
	50k < 80k			2.56	1.76, 3.73
	20k < 50k			3.97	2.78, 5.66
	Less than 20k			6.56	4.42, 9.72
Remoteness	Major city			1	
	Inner regional			1.10	0.84, 1.43
	Outer regional			1.04	0.75, 1.44
	Remote/Very remote			1.55	0.98, 2.44
Random parameters		Est.		Est.	
ICC (%)		2.2%		0.2%	
MOR		1.30		1.09	
P-value for the interaction between LGA level income inequality and LGA level mean weekly household income (p<0.001)					
Mean weekly household income		Income Inequality		OR	95% CI
	High	Low		1	
		Medium		0.81	0.51, 1.29
		High		0.58	0.37, 0.91
	Medium	Low		1	
		Medium		0.86	0.59, 1.26
		High		1.05	0.59, 1.88
	Low	Low		1	
		Medium		0.92	0.60, 1.39
		High		0.56	0.18, 1.74

Model 1: Unadjusted; Model 2: Adjusted for age, sex, LGA level mean income, household income and remoteness; ICC: Intra-class Coefficient, MOR: Median Odds Ratio, Est.: Estimate; OR: Odds ratio

Model 1 showed that individuals in LGAs with the highest tertile of Gini had relative odds of 0.77 for having poor self-rated oral health compared to those in LGAs of lowest tertile of Gini (Table 3). This association did not remain significant after inclusion of covariates age, sex, LGA-level mean household weekly income, and household income (Model 2, Table 3). The MOR for poor self-rated oral health was close to 1, and after inclusion of age, sex, household income, and LGA level mean income in the final model MOR was 1.04. Residents of middle income, medium inequality LGAs had relatively lower odds (OR: 0.80; 95% CI: 0.61, 1.05) of poor self-rated oral health than their counterparts living in low inequality LGAs. While residents of middle income, high inequality LGAs had relatively higher odds (OR: 1.35; 95% CI: 0.91, 2.01) (Table 3).

**Table 3:** Multilevel logistic regression analysis for the association between LGA level income inequality and poor self-rated oral health (N of Areas = 435; N of individuals = 5,165).

		Model 1		Model 2	
	Categories	OR	95% CI	OR	95% CI
Income Inequality (Gini)	Low	1		1	
	Medium	0.93	0.79, 1.10	0.95	0.80, 1.13
	High	0.77	0.65, 0.91	0.92	0.74, 1.14
Mean weekly household income	High			1	
	Medium			1.10	0.91, 1.33
	Low			1.29	1.02, 1.65
Age	1-year change			1.00	0.99, 1.00
Sex	Male			1	
	Female			0.79	0.68, 0.91
Household Income	$100K and above			1	
	80K < 100k			1.39	1.07, 1.81
	50k < 80k			1.78	1.44, 2.21
	20k < 50k			2.62	2.12, 3.22
	Less than 20k			4.08	3.13, 5.31
Remoteness	Major city			1	
	Inner regional			0.87	0.71, 1.06
	Outer regional			0.94	0.74, 1.19
	Remote/Very remote			1.46	1.06, 2.01
Random parameters		Est.		Est.	
ICC (%)		<0.1%		0.4%	
MOR		~1.00		1.04	
P-value for the interaction between LGA level income inequality and LGA level mean weekly household income (p = 0.15)					
Mean weekly household income		Income Inequality		OR	95% CI
	High	Low		1	
		Medium		1.33	0.95, 1.87
		High		1.04	0.75, 1.45
	Medium	Low		1	
		Medium		0.80	0.61, 1.05
		High		1.35	0.91, 2.01
	Low	Low		1	
		Medium		0.97	0.66, 1.44
		High		1.66	0.67, 4.10

Model 1: Unadjusted; Model 2: Adjusted for age, sex, LGA level mean income, household income and remoteness; ICC: Intra-class Coefficient, MOR: Median Odds Ratio, Est.: Estimate; OR: Odds ratio

**Fig 2:**
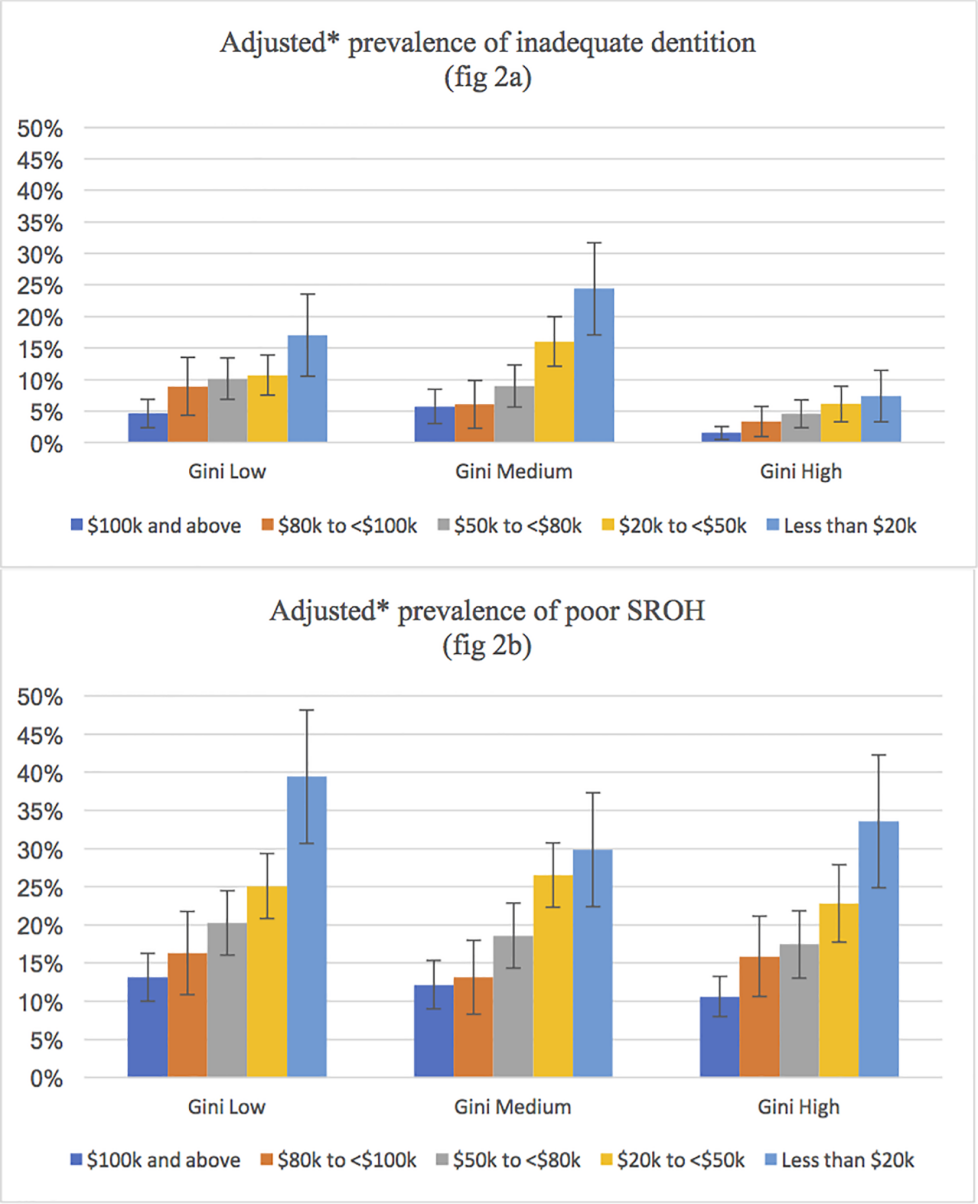
Adjusted prevalence of inadequate dentition (Fig 2A), and poor self-rated oral health (Fig 2B) according to household income and LGA level income inequality (*adjusted for age, sex, educational attainment, LGA level mean weekly household income and remoteness).

The adjusted prevalence of inadequate dentition by household income and LGA level income inequality showed that although there was an overall lower prevalence of inadequate dentition within the LGAs with highest tertile of Gini, a clear stepwise gradient with household income was observed in this group. On the other hand, a marked increase in the prevalence of inadequate dentition within the groups of low and medium inequality was observed at the household income levels of less than $20,000 and $20,000 - $50,000 groups, respectively (Fig 2A). There were no differences in household income according to inequality for the outcome of poor self-rated oral health (Fig 2B).

Changes in estimates on sequential adjustment of covariates (age, sex + LGA-level mean is also presented in Supporting files S1 Appendix, S2 & S3 Tables. Findings from the sensitivity analyses confirmed the robustness of findings (Supporting files: S2 Appendix and S4, S5, S6, S7 & S8 Tables).

## Discussion

Higher area-level income inequality was found to be associated with lower inadequate dentition at the individual level among Australian adults, but no association was present for poor self-rated oral health. The share of the individual-level variation in the outcome of inadequate dentition was higher at the LGA-level compared to the outcome of poor self-rated oral health. Stratified analysis confirmed that the association between higher income inequality and lower inadequate dentition was limited to areas with high mean income. Oral health was poorer at lower levels of household income. Differences in gradients of oral health by household income were observed across levels of LGA income inequality for inadequate dentition, but not for self-rated oral health.

This study has several strengths. It is the first assessment of the association between area-level income inequality and oral health within Australia using a robust methodology on a nationally representative dataset; weighted according to Australian Census of Population and Housing 2011 [36]. The multilevel analytical technique has advantages in testing associations between area-level income inequality and health outcomes as it allows accounting for potential confounding at both area and individual level [32]. To the best of our knowledge this study is the first within Australia to apply the multilevel technique to test the association between income inequality and health. The data from NDTIS 2013 had a wide coverage with individuals from 78% (n = 435/561) of LGAs in Australia. This is recognized as an advantage when conducting the multilevel analysis [44–46]. Due to the difference of timing between Australian Census 2011, and NDTIS 2013, a natural lag time of two years between the exposure area-level income inequality and the oral health outcomes was present. It is stated within the literature that societal factors such as income inequality may not have an instantaneous effect on health [47], and therefore this was an added strength of the study. Multiple sensitivity analyses were performed to confirm the robustness of our findings in Australian context. Testing associations of area-level income inequality with both a subjective (self-rated oral health) and objective measure (inadequate dentition) of oral health was an additional strength of this study [14]. Finally, this study tested both the independent and combined associations of area-level income inequality and mean income on oral health outcomes at the individual level as well as the differences in income gradients in these outcomes according to area level income inequality in the Australian context following recent suggestions within the literature [30, 35]. Therefore, the study adds to the empirical evidence on the theorized interdependencies between different dimensions of income at an individual and societal level on health, raised within the literature [30, 35].

Some limitations were also there. Given that the information regarding the temporal sequence between oral health outcomes and the exposure of income inequality was not available, causal inferences cannot be made from the current study. There were missing values for the outcomes and co-variates due to which all dentate participants within NDTIS 2013 could not be analyzed leading to a reduction in sample size. Majority of the missing values were identified for the variable of household income that can potentially lead to selection bias and affect the generalizability of the findings. Only dentates were analyzed in the current study and edentates may present as the most severe form of tooth loss. However, a continuing trend of fall in edentulism in Australia has been reported and a low prevalence of edentulism (4.7%) was confirmed in the NDTIS 2013 [36]. Tertiles of LGA-level Gini coefficient were used in the analysis to draw relative comparisons among individuals according to area-level income inequality. However, the intervals of these tertiles were not equal in size. A sensitivity analysis confirmed that the observed associations between LGA-level income inequality and inadequate dentition was present when categorization of Gini coefficients were alternatively derived from k-cluster analysis (Supporting file: S7 Table).

Most studies on area-level income inequality and health have either shown no associations or that higher income inequality is associated with worse health outcomes [1, 6]. The finding of higher area-level income inequality to be associated with lower inadequate dentition at the individual level is conflicting with this literature. However, this study is not the first to report the association in the direction opposite to proposed hypothesis. An Australian ecological study has also reported that higher income inequality at the LGA level was associated with lower alcohol-attributed deaths and hospitalization [28]. Higher income inequality at a small area level has also been shown to be associated with lower mortality in Belgium [48] and Switzerland [49], lower adverse birth outcomes and better self-perceived health in Canada [50, 51], and USA [52], and better mental health outcomes in Wales [53]. No previous study on oral health outcomes has reported higher area-level income inequality to be associated with better oral health outcomes [7]. A study from Wales reported an association between higher area-level income inequality and lower common mental disorders only in low deprivation neighborhoods [53], which is consistent with our findings of an inverse association between area-level income inequality and inadequate dentition limited to LGAs with the highest mean income.

Number of possible explanations exist for the differences in the presence and direction of associations between income inequality and the two outcomes in the Australian context. There is a strong potential of residual confounding due to both measured and unmeasured covariates. A detailed examination of spatial characteristics of income inequality among working age males at the Statistical Local Area (SLA) level (similar in geography to LGAs) in Australia has revealed two interesting patterns. First, income inequality at the LGA level is positively correlated with average income within major Australian cities. Therefore, residual confounding due to LGA-level mean weekly household income can possibly drive the counterintuitive findings for inadequate dentition. A sensitivity analysis showed attenuation in odds ratio for inadequate dentition (OR 0.68; 95% CI: 0.50, 0.92) when LGA-level mean weekly household income and household income were alternatively included on a continuous scale for adjustment in multilevel multivariable logistic regression model (S8 Table). However, the 95% confidence intervals did not include null. Second, in most Australian cities, income inequality is much higher in the more heterogeneous inner-city areas compared to the outer regions of the cities that are more homogenous areas with low average incomes [54]. The current study also found that the inverse association between area-level income inequality and inadequate dentition was limited to areas with high mean income. Inadequate dentition is an outcome of tooth loss that is a cumulative outcome of an individual’s lifetime exposure to dental disease and utilization of dental care. At a country level, studies have shown that utilization of dental care is inversely associated with income inequality [55]. The dental care system in Australia comprises a combination of private and public sectors and the majority of dental services for adults are provided through the private sector. The state and territory governments provide free or subsidized dental care to those who hold an Australian Government concession card. At the LGA level within Australia, it is more likely that area-level mean income rather than inequality is likely to drive access to dental care. Studies have examined access to dental care as mediators between the area-level income inequality and oral health outcomes in the USA and Brazil consistent with the neo-material pathway [7, 16, 56]. Due to the lack of available data on dentist to population ratio at the LGA level within Australia, the role of access to dental services in the association between LGA-level income inequality and inadequate dentition could not be examined in this study. Therefore, potential unmeasured/residual confounding may explain the counterintuitive findings for inadequate dentition.

A geographic phenomenon may also explain the findings for inadequate dentition. The association between income inequality and health outcomes is sensitive to the level of geographic aggregation at which the association is tested [6]. Modifiable areal unit problem is a phenomena where societal exposures likely vary based on the definition of the geographic scale selected as well as zonation areas even when one scale is selected [57]. Therefore, the observed association can vary at other levels of geographic aggregation and future studies should confirm the current findings at different levels of geographic aggregation. Additionally, socio-epidemiologic theoretical pathways that are proposed to explain the association between area-level income inequality and health outcomes hinge upon income inequality as a marker of social inequality [33, 58]. Large differences are reported in wealth inequality (Gini for household net worth in 2013–14 was 0.605) and income inequality (Gini = 0.333 for equivalised disposable household income) at the national level [59]. Therefore, structural differences between small areas in Australia and other countries could lead to the observation of lower inadequate dentition at higher levels of LGA-level income inequality. It is beyond the scope of the current study to examine whether income inequality at LGA level can well capture underlying class relations and the degree of social stratification [33, 60]. Future studies could also investigate associations with area-level income inequality and oral health among adolescents and children as the rates of child poverty are high in Australia. It may be possible the income inequality has different impacts on different population groups according to age [38].

The current study found differences in income gradients in the prevalence of inadequate dentition between LGAs at different levels of income inequality. Overall, the prevalence of both the oral health outcomes (poor self-rated oral health and inadequate dentition) was higher with decreasing household income in each group. While there was a clear stepwise gradient in the prevalence of inadequate dentition in the LGAs with high Gini reflecting overall susceptibility towards inadequate dentition across income groups, individuals at lower household incomes were more vulnerable towards inadequate dentition in LGAs with low and medium Gini. This finding substantiates the need to investigate slope effects of area-level income inequality on the association between individual income and health in conjunction with the average effects that examine overall effect of income inequality on health [35].

In conclusion, current findings highlight important contextual differences at small area level between other countries and Australia. Hence, generalization of evidence on the negative impact of a societal determinant (income inequality) on health from one context to other is inappropriate.

## Supporting information

S1 Appendix Supporting information for multilevel multivariable regression models. (DOCX)

S2 Appendix Sensitivity analyses. (DOCX)

S1 Fig Sample flowchart. (DOCX)

S1 Table Descriptive characteristics of the sample according to different sample groups. (DOCX)

S2 Table Multilevel logistic regression analysis for the association between LGA level income inequality and inadequate dentition (No. of Areas = 428; N of individuals = 4,768). (DOCX)

S3 Table Multilevel logistic regression analysis for the association between LGA level income inequality and poor self-rated oral health (N of Areas = 435; N of individuals = 5,165). (DOCX)

S4 Table Sensitivity analysis (sensitivity analysis -1) to investigate differences in the associations of income inequality and oral health outcomes by cluster size. (DOCX)

S5 Table Sensitivity analysis (sensitivity analysis-2) to investigate variations in the associations between area-level income inequality and inadequate dentition and poor self-rated oral health among LGAs within Australia. (DOCX)

S6 Table Sensitivity analysis (sensitivity analysis-3) to investigate variations in the associations between area-level income inequality and inadequate dentition and poor self-rated oral health after adjusting for additional covariates. (DOCX)

S7 Table Sensitivity analysis (sensitivity analysis-4) to investigate for variation in the association between LGA-level income inequality and inadequate dentition according to a categorization of LGA-level income inequality derived through k-cluster analysis. (DOCX)

S8 Table Sensitivity analysis (sensitivity analysis-5) to investigate for residual confounding by LGA-level mean household income and household income. (DOCX)

## References

[1] Pickett KE, Wilkinson RG. Income inequality and health: a causal review. Soc Sci Med. 2015;128:316–26. Epub 2015/01/13. doi: 10.1016/j.socscimed.2014.12.031 . 25577953 10.1016/j.socscimed.2014.12.031

[2] Macinko JA, Shi L, Starfield B, Wulu JT. Income inequality and health: a critical review of the literature. Medical Care Research and review: MCRR. 2003;60(4):407 doi: 10.1177/1077558703257169 14677219 10.1177/1077558703257169

[3] Lynch J, Smith GD, Harper S, Hillemeier M, Ross N, Kaplan GA, et al Is Income Inequality a Determinant of Population Health? Part 1. A Systematic Review. The Milbank Quarterly. 2004;82(1):5–99. doi: 10.1111/j.0887-378X.2004.00302.x 15016244 10.1111/j.0887-378X.2004.00302.x PMC2690209

[4] Subramanian SV, Blakely T, Kawachi I. Income inequality as a public health concern: where do we stand? Commentary on "Is exposure to income inequality a public health concern?". Health Serv Res. 2003;38(1 Pt 1):153–67. Epub 2003/03/26. doi: 10.1111/1475-6773.00110 ; PubMed Central PMCID: PMCPMC1360879. 12650386 10.1111/1475-6773.00110 PMC1360879

[5] Kondo N, Sembajwe G, Kawachi I, van Dam RM, Subramanian SV, Yamagata Z. Income inequality, mortality, and self rated health: meta-analysis of multilevel studies. BMJ. 2009;339(7731):1178–81. 10.1136/bmj.b4471 PMC2776131 19903981

[6] Wilkinson RG, Pickett KE. Income inequality and population health: a review and explanation of the evidence. Soc Sci Med. 2006;62(7):1768–84. Epub 2005/10/18. doi: 10.1016/j.socscimed.2005.08.036 . 16226363 10.1016/j.socscimed.2005.08.036

[7] Singh A, Harford J, Schuch HS, Watt RG, Peres MA. Theoretical basis and explanation for the relationship between area-level social inequalities and population oral health outcomes–A scoping review. SSM—Population Health. 2016;2:451–62. http://dx.doi.org/10.1016/j.ssmph.2016.06.001. 29349160 10.1016/j.ssmph.2016.06.001 PMC5757950

[8] Kassebaum N, Smith A, Bernabé E, Fleming T, Reynolds A, Vos T, et al Global, Regional, and National Prevalence, Incidence, and Disability-Adjusted Life Years for Oral Conditions for 195 Countries, 1990–2015: A Systematic Analysis for the Global Burden of Diseases, Injuries, and Risk Factors. J Dent Res. 2017;96(4):380–7. doi: 10.1177/0022034517693566 28792274 10.1177/0022034517693566 PMC5912207

[9] Listl S, Galloway J, Mossey PA, Marcenes W. Global Economic Impact of Dental Diseases. J Dent Res. 2015;94(10):1355–61. doi: 10.1177/0022034515602879 . 26318590 10.1177/0022034515602879

[10] Sheiham A. Oral health, general health and quality of life. Bulletin of the World Health Organization. 2005;83(9):644 Epub 2005/10/08. doi: /S0042-96862005000900004 ; PubMed Central PMCID: PMCPMC2626333. 16211151 PMC2626333

[11] Haag DG, Peres KG, Balasubramanian M, Brennan DS. Oral Conditions and Health-Related Quality of Life: A Systematic Review. Journal of dental research. 2017:0022034517709737. doi: 10.1177/0022034517709737 28581891 10.1177/0022034517709737

[12] Hobdell M, Petersen P, Clarkson J, Johnson N. Global goals for oral health 2020. International Dental Journal. 2003;53(5):285–8. doi: 10.1111/j.1875-595X.2003.tb00761.x 14560802 10.1111/j.1875-595x.2003.tb00761.x

[13] Gerritsen AE, Allen PF, Witter DJ, Bronkhorst EM, Creugers NH. Tooth loss and oral health-related quality of life: a systematic review and meta-analysis. Health and Qquality of Life Outcomes. 2010;8:126 Epub 2010/11/06. doi: 10.1186/1477-7525-8-126 ; PubMed Central PMCID: PMCPMC2992503. 21050499 10.1186/1477-7525-8-126 PMC2992503

[14] Locker D, Mscn EW, Jokovic A. What do older adults' global self-ratings of oral health measure? J Public Health Dent. 2005;65(3):146–52. Epub 2005/09/21. . 16171259 10.1111/j.1752-7325.2005.tb02804.x

[15] Bernabe E, Marcenes W. Income inequality and tooth loss in the United States. J Dent Res. 2011;90(6):724–9. Epub 2011/04/22. doi: 10.1177/0022034511400081 . 21508433 10.1177/0022034511400081

[16] Celeste RK, Nadanovsky P, Ponce de Leon A, Fritzell J. The individual and contextual pathways between oral health and income inequality in Brazilian adolescents and adults. Soc Sci Med. 2009;69(10):1468–75. Epub 2009/09/22. doi: 10.1016/j.socscimed.2009.08.005 . 19765876 10.1016/j.socscimed.2009.08.005

[17] Goulart Mde A, Vettore MV. Is the relative increase in income inequality related to tooth loss in middle-aged adults? J Public Health Dent. 2016;76(1):65–75. Epub 2015/08/01. doi: 10.1111/jphd.12113 . 26228934 10.1111/jphd.12113

[18] Vettore MV, Efhima S, Machuca C, de Almeida Lamarca G. Income inequality and traumatic dental injuries in 12-years old children: a multi-level analysis. Dental traumatology: official publication of International Association for Dental Traumatology. 2017 Epub 2017/05/26. doi: 10.1111/edt.12350 . 28544700 10.1111/edt.12350

[19] Aida J, Kondo K, Kondo N, Watt RG, Sheiham A, Tsakos G. Income inequality, social capital and self-rated health and dental status in older Japanese. Soc Sci Med. 2011;73(10):1561–8. Epub 2011/10/11. doi: 10.1016/j.socscimed.2011.09.005 . 21982631 10.1016/j.socscimed.2011.09.005

[20] Peres MA, Peres KG, Antunes JL, Junqueira SR, Frazao P, Narvai PC. The association between socioeconomic development at the town level and the distribution of dental caries in Brazilian children. Revista panamericana de salud publica = Pan American journal of public health. 2003;14(3):149–57. Epub 2003/12/05. . 14653902 10.1590/s1020-49892003000800001

[21] Celeste RK, Fritzell J, Nadanovsky P. The relationship between levels of income inequality and dental caries and periodontal diseases. Cadernos de saude publica. 2011;27(6):1111–20. Epub 2011/06/29. . 21710008 10.1590/s0102-311x2011000600008

[22] Chalub LL, Martins CC, Ferreira RC, Vargas AM. Functional Dentition in Brazilian Adults: An Investigation of Social Determinants of Health (SDH) Using a Multilevel Approach. PloS one. 2016;11(2):e0148859 doi: 10.1371/journal.pone.0148859 ; PubMed Central PMCID: PMC4749636. 26862892 10.1371/journal.pone.0148859 PMC4749636

[23] Dabla-Norris E, Kochhar K, Suphaphiphat N, Ricka F, Tsounta E. Causes and Consequences of Income Inequality: A Global Perspective. International Monetary Fund, 2015.

[24] Piketty T. Capital in the Twenty-First Century: CAMBRIDGE, MASSACHUSETTS LONDON, ENGLAND: Harvard University Press; 2014.

[25] Wilkinson R, Pickett KE. The spirit level: why more equal societies almost always do better Pickett K, editor. London: Allen Lane; 2009.

[26] Kawachi I, Kennedy BP. The relationship of income inequality to mortality: does the choice of indicator matter. Soc Sci Med. 1997;45(7):1121–7. 9257403 10.1016/s0277-9536(97)00044-0

[27] Fletcher M, Guttmann B. Income Inequality in Australia. Canberra: Australian Government: The Treasury, 2013.

[28] Dietze PM, Jolley DJ, Chikritzhs TN, Clemens S, Catalano P, Stockwell T. Income inequality and alcohol attributable harm in Australia. BMC Public Health. 2009;9(70):1–9. doi: Artn 70 doi: 10.1186/1471-2458-9-70 PubMed PMID: WOS:000265065000001. 19239715 10.1186/1471-2458-9-70 PMC2658667

[29] Bechtel L, Lordan G, Rao DS. Income inequality and mental health—empirical evidence from Australia. Health Econ. 2012;21 Suppl 1:4–17. Epub 2012/05/11. doi: 10.1002/hec.2814 . 22556000 10.1002/hec.2814

[30] Rambotti S. Recalibrating the spirit level: An analysis of the interaction of income inequality and poverty and its effect on health. Soc Sci Med. 2015;139:123–31. Epub 2015/03/03. doi: 10.1016/j.socscimed.2015.02.026 . 25726520 10.1016/j.socscimed.2015.02.026

[31] Sanders AE, Turrell G, Slade GD. Affluent neighborhoods reduce excess risk of tooth loss among the poor. J Dent Res. 2008;87(10):969–73. Epub 2008/09/24. doi: 10.1177/154405910808701006 . 18809753 10.1177/154405910808701006

[32] Subramanian SV, Kawachi I. Income inequality and health: what have we learned so far? Epidemiol Rev. 2004;26:78–91. Epub 2004/07/06. doi: 10.1093/epirev/mxh003 . 15234949 10.1093/epirev/mxh003

[33] Muntaner C, Lynch J. Income Inequality, Social Cohesion, and Class Relations: A critique of Wilkinson's Neo-Durkheimian research programme. International Journal of Health Services. 1999;29(1):59–81. doi: 10.2190/G8QW-TT09-67PL-QTNC 10079398 10.2190/G8QW-TT09-67PL-QTNC

[34] Judge K, Mulligan JA, Benzeval M. The relationship between income inequality and population health. Soc Sci Med. 1998;47(7):983 9722117 10.1016/s0277-9536(98)00132-4

[35] Truesdale BC, Jencks C. The Health Effects of Income Inequality: Averages and Disparities. Annu Rev Public Health. 2016;37(1):413–30. doi: 10.1146/annurev-publhealth-032315-021606 . 26735427 10.1146/annurev-publhealth-032315-021606

[36] AIHW [Australian Institute of Health and Welfare]. National Dental Telephone Interview Survey 2013. Canberra: Australian Institute of Health and Welfare http://meteor.aihw.gov.au/content/index.phtml/itemId/629709, 2016.

[37] Luzzi L, Harford J. Financial burden of dental care among Australian children. Aust Dent J. 2014;59(2):268–72. Epub 2014/05/28. doi: 10.1111/adj.12181 . 24861406 10.1111/adj.12181

[38] Dorling D, Mitchell R, Pearce J. The global impact of income inequality on health by age: an observational study. BMJ. 2007;335(7625):873 Epub 2007/10/24. doi: 10.1136/bmj.39349.507315.DE ; PubMed Central PMCID: PMCPMC2043415. 17954512 10.1136/bmj.39349.507315.DE PMC2043415

[39] Association ALG. About ALGA: Australian Local Government Association; 2014 [cited 2016 17 December]. Available from: http://alga.asn.au/?ID=42&Menu=41,81.

[40] ABS. Information Paper Converting Data to the Australian Statistical Geography Standard. Canberra: Australian Bureau of Statistics, 2012 ABS Catalogue No. 1216.0.55.004.

[41] Fleming DA, Measham TG. Income Inequality across Australian Regions during the Mining Boom: 2001–11. Australian Geographer. 2015;46(2):203–16. doi: 10.1080/00049182.2015.1020596

[42] ABS. Census Dictionary—Australia. Canberra: Australian Bureau of Statistics, 2011.

[43] Merlo J, Chaix B, Ohlsson H, Beckman A, Johnell K, Hjerpe P, et al A brief conceptual tutorial of multilevel analysis in social epidemiology: using measures of clustering in multilevel logistic regression to investigate contextual phenomena. J Epidemiol Community Health. 2006;60(4):290–7. doi: 10.1136/jech.2004.029454 PubMed PMID: WOS:000235971500003. 16537344 10.1136/jech.2004.029454 PMC2566165

[44] Snijders TAB. Power and sample size in multilevel modeling In: Everitt BS, Howell DC, editors. Encyclopedia of Statistics in Behavioral Science. 3 Chicester: Wiley; 2005 p. 1570–3.

[45] Rabe-Hesketh S, Skrondal A. Variance Component Models In: Rabe-Hesketh S, Skrondal A, editors. Multilevel and Longitudinal Modeling Using Stata: Second Edition Texas: Stata Press; 2008 p. 62.

[46] Theall KP, Scribner R, Broyles S, Yu QZ, Chotalia J, Simonsen N, et al Impact of small group size on neighbourhood influences in multilevel models. J Epidemiol Community Health. 2011;65(8):688–95. doi: 10.1136/jech.2009.097956 PubMed PMID: WOS:000292318000010. 20508007 10.1136/jech.2009.097956 PMC3706628

[47] Blakely TA, Woodward AJ. Ecological effects in multi-level studies. J Epidemiol Community Health. 2000;54(5):367–74. doi: 10.1136/jech.54.5.367 ; PubMed Central PMCID: PMC1731678. 10814658 10.1136/jech.54.5.367 PMC1731678

[48] Lorant V, Thomas I, Deliege D, Tonglet R. Deprivation and mortality: the implications of spatial autocorrelation for health resources allocation. Soc Sci Med. 2001;53(12):1711–9. Epub 2002/01/05. . 11762895 10.1016/s0277-9536(00)00456-1

[49] Clough-Gorr KM, Egger M, Spoerri A. A Swiss paradox? Higher income inequality of municipalities is associated with lower mortality in Switzerland. Eur J Epidemiol. 2015;30(8):627–36. Epub 2015/01/21. doi: 10.1007/s10654-015-9987-7 . 25600296 10.1007/s10654-015-9987-7

[50] Hou F, Myles J. Neighbourhood inequality, neighbourhood affluence and population health. Soc Sci Med. 2005;60(7):1557–69. Epub 2005/01/18. doi: 10.1016/j.socscimed.2004.08.033 . 15652687 10.1016/j.socscimed.2004.08.033

[51] Auger N, Giraud J, Daniel M. The joint influence of area income, income inequality, and immigrant density on adverse birth outcomes: a population-based study. BMC Public Health. 2009;9:237 Epub 2009/07/16. doi: 10.1186/1471-2458-9-237 ; PubMed Central PMCID: PMCPMC2714302. 19602256 10.1186/1471-2458-9-237 PMC2714302

[52] Wen M, Browning CR, Cagney KA. Poverty, affluence, and income inequality: neighborhood economic structure and its implications for health. Soc Sci Med. 2003;57(5):843–60. Epub 2003/07/10. . 12850110 10.1016/s0277-9536(02)00457-4

[53] Fone D, Greene G, Farewell D, White J, Kelly M, Dunstan F. Common mental disorders, neighbourhood income inequality and income deprivation: small-area multilevel analysis. The British Journal of Psychiatry. 2013;202(4):286–93. doi: 10.1192/bjp.bp.112.116178 23470284 10.1192/bjp.bp.112.116178 PMC3613720

[54] Bradbury B. Spatial inequality of Australian men's incomes, 1991 to 2011. Australian Labour Market Research Conference, Canberra; Canberra2017.

[55] Bhandari B, Newton JT, Bernabe E. Income Inequality and Use of Dental Services in 66 Countries. J Dent Res. 2015;94(8):1048–54. Epub 2015/05/23. doi: 10.1177/0022034515586960 . 25994178 10.1177/0022034515586960

[56] Bernabe E, Marcenes W. Income inequality and tooth loss in the United States. J Dent Res. 2011;90(6):724–9. Epub 2011/04/22. doi: 10.1177/0022034511400081 . 21508433 10.1177/0022034511400081

[57] Duncan DT, Kawachi I, Subramanian SV, Aldstadt J, Melly SJ, Williams DR. Examination of how neighborhood definition influences measurements of youths' access to tobacco retailers: a methodological note on spatial misclassification. American journal of epidemiology. 2014;179(3):373–81. Epub 2013/10/24. doi: 10.1093/aje/kwt251 ; PubMed Central PMCID: PMCPMC3895093. 24148710 10.1093/aje/kwt251 PMC3895093

[58] Goldthorpe JH. Analysing Social Inequality: A Critique of Two Recent Contributions from Economics and Epidemiology. European Sociological Review. 2010;26(6):731–44. doi: 10.1093/esr/jcp046

[59] ABS. Household Income and Income Distribution, Australia, 1994–95 to 2013–14. In: Statistics Australian Bureau of Statistics, editor. Canberra2015.

[60] Wilkinson RG. Income inequality, social cohesion, and health: clarifying the theory—a reply to Muntaner and Lynch. Int J Health Serv. 1999;29(3):525–43. Epub 1999/08/18. doi: 10.2190/3QXP-4N6T-N0QG-ECXP . 10450545 10.2190/3QXP-4N6T-N0QG-ECXP

