# Supplementary material for: Area-level income inequality and oral health among Australian adults—A population-based multilevel study
Source: PLoS One. 2018 Jan 24;13(1):e0191438. doi: 10.1371/journal.pone.0191438 (PMC5783384; doi:10.1371/journal.pone.0191438)
Supplement: S1 Appendix — (DOCX) [file pone.0191438.s001.docx]

**S1. Appendix**

**Collinearity**

Index of Relative Socioeconomic Advantage and Disadvantage (IRSAD) score is a measure of Socio Economic Indexes for Areas (SEIFA), an area-level composite index that summarises information about economic and social conditions of people and households within an LGA including both relative advantage and disadvantage measures. IRSAD scores were obtained from the Australian Census of Population and Housing 2011 and converted into tertiles. Pearson’s correlation coefficients were obtained to check for collinearity between LGA-level Gini coefficients and LGA-level weekly mean household income and IRSAD scores. LGA-level Gini and LGA-level mean household weekly income had significant but weak correlations (ρ=0.29, p<0.001). Similarly, significant but weak correlations (ρ=0.12, p=0.008) were observed between LGA-level Gini and IRSAD scores. Significantly strong correlations were noted between LGA-level mean household weekly income and IRSAD scores (ρ=0.79, p<0.001).

**Sequential adjustment for covariates**

**Analysis**

Multilevel multivariable regression models with random intercept and fixed slopes were fitted to test associations between income inequality (tertiles of Gini coefficient) and the two oral health outcomes at individual level. Model 1 represented null model with no explanatory variables. Model 2 estimated the unadjusted association between the tertiles of Gini coefficients for LGAs and the two outcomes. A sequential adjustment of covariates was then carried out as follows: model 3 adjusted for age and sex, model 4 for LGA-level weekly mean household income, and model 5 for household income and geographic remoteness. All the models are presented below while only fully adjusted models are presented in the text of the manuscript.

**Results**

Unadjusted estimates obtained from model 1 showed that individuals in the most unequal LGAs had relative odds of 0.59 for inadequate dentition compared to individuals in the least unequal LGAs (S2 Table). Adding age and sex in model 2 reduced the relative odds to 0.43, while the inclusion of LGA-level mean household weekly income increased it to 0.58. After adjusting for individual age, sex and household income, and LGA-level mean household weekly income, individuals in most unequal LGAs had relative odds of 0.64 of having inadequate dentition, with LGAs in the lowest tertile of Gini at reference. (S2 Table).

Model 1 showed that individuals in LGAs with the highest tertile of Gini had relative odds of 0.77 for having poor self-rated oral health compared to those in LGAs of lowest tertile of Gini (S3 Table). The inclusion of age and sex in model 2 attenuated the odds ratio, but not markedly. LGA-level mean household weekly income attenuated this association and the odds ratio was 0.89 and non-significant (model 3).
