# Supplementary material for: Area-level income inequality and oral health among Australian adults—A population-based multilevel study
Source: PLoS One. 2018 Jan 24;13(1):e0191438. doi: 10.1371/journal.pone.0191438 (PMC5783384; doi:10.1371/journal.pone.0191438)
Supplement: S2 Appendix — (DOCX) [file pone.0191438.s002.docx]

**S2. Appendix**

Five different sensitivity analyses were performed to confirm the robustness of findings:

1. The first sensitivity analysis was performed to investigate if differences in associations exist according to cluster sizes (low observations in each LGA). Two cut offs for sample sizes were tested following suggestions from the literature [1, 2] –minimum of two individuals per LGA (excluding singletons– LGAs with only one individual) and a minimum of five individuals per LGA (S4 Table).
2. The second sensitivity analysis tested whether the observed associations between area-level income inequality and oral health outcomes at the individual level varied among LGAs. Measures of 80% Interval Odds Ratio (IOR) and Proportion of Opposite Odds Ratio (POOR) that are estimated using the specific regression coefficient and variance attributed to LGAs from each regression model informed the degree of variations in the associations (S5 Table) [3].

| *80% IOR  (If includes 1 then some areas have association in opposite direction to overall odds ratio) | 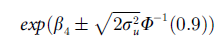 |
| --- | --- |
| #Proportion of Opposite Odds Ratio (POOR)  (Values extend from 0% to 50%. A POOR of 0% means all ORs have the same sign. A  POOR of 50% means that half of the ORs are of the opposite sign and so the association is very heterogeneous.) | 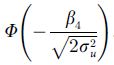 |

1. The third sensitivity analysis confirmed if the observed associations between income inequality and oral health outcomes were robust to adjustment of variables (education and tertiles of LGA-level IRSAD) where there is a lack of clarity and consensus on their role in the association between income inequality and the two oral health outcomes in the Australian context (S6 Table).
2. The final sensitivity analysis examined the association between LGA-level income inequality and inadequate dentition with the categorization of LGA-level Gini coefficients derived through k-cluster analysis (S7 Table).
3. A sensitivity analysis was performed to examine the association between LGA-level income inequality and inadequate dentition under different categories of LGA-level mean income (deciles) and 8 categories of household income. This analysis examined the potential of residual confounding by area-level, and household-level, measures of income (S8 Table).

References

1. Rabe-Hesketh S, Skrondal A. Variance Component Models. In: Rabe-Hesketh S, Skrondal A, editors. Multilevel and Longitudinal Modeling Using Stata: Second Edition. Texas: Stata Press; 2008. p. 62.

2. Theall KP, Scribner R, Broyles S, Yu QZ, Chotalia J, Simonsen N, et al. Impact of small group size on neighbourhood influences in multilevel models. Journal of epidemiology and community health. 2011;65(8):688-95. doi: 10.1136/jech.2009.097956. PubMed PMID: WOS:000292318000010.

3. Merlo J, Wagner P, Ghith N, Leckie G. An Original Stepwise Multilevel Logistic Regression Analysis of Discriminatory Accuracy: The Case of Neighbourhoods and Health. PloS one. 2016;11(4). doi: ARTN e0153778

10.1371/journal.pone.0153778. PubMed PMID: WOS:000374976200036.
