# Supplementary material for: Area-level income inequality and oral health among Australian adults—A population-based multilevel study
Source: PLoS One. 2018 Jan 24;13(1):e0191438. doi: 10.1371/journal.pone.0191438 (PMC5783384; doi:10.1371/journal.pone.0191438)
Supplement: S1 Fig — (DOCX) [file pone.0191438.s003.docx]

S1 Fig. Sample flowchart

Excluded: 401 missing values for number of teeth

Analyzed sample Inadequate dentition: 4,768

Analyzed sample Self rated oral health: 5,165

Excluded: 4 missing values for self-rated oral health

Dentates: 5,169

Excluded: 307 edentates (non-relevant sample)

))

Excluded: 83 could not be allocated to LGAs

Full cases allocated to LGA: 5,476

Excluded: 781 missing for household income

Full cases with individual income: 5,559

Interviewed: 6,340
