# Supplementary material for: Area-level income inequality and oral health among Australian adults—A population-based multilevel study
Source: PLoS One. 2018 Jan 24;13(1):e0191438. doi: 10.1371/journal.pone.0191438 (PMC5783384; doi:10.1371/journal.pone.0191438)
Supplement: S1 Table — (DOCX) [file pone.0191438.s004.docx]

| S1. Table. Descriptive characteristics of the sample according to different sample groups | | | | | | |
| --- | --- | --- | --- | --- | --- | --- |
| Characteristics | Categories | Australian Population Census (2011) | Interviewed-NDTIS (n=6,340)  (%) | Dentates (n=5,978) | Full Case  SROH (n=5,165) | Full Case  Inadequate Dentition (n=4,678) |
| Sex | Male | 49.7 | 49.3 | 49.6 | 50.6 | 48.5 |
|  | Female | 50.3 | 50.7 | 50.4 | 49.4 | 51.5 |
| Age | 18-34 | 28.04 (15-34 years) | 31.5 | 33.0 | 30.1 | 28.7 |
|  | 35-54 | 27.8 | 35.2 | 36.5 | 38.7 | 39.1 |
|  | 55-74 | 19.01 | 25.1 | 24.2 | 25.1 | 26.0 |
|  | 75 and above | 6.3 | 8.2 | 6.3 | 6.1 | 6.1 |
| Household Income | $100K and above |  | 27.4 | 28.6 | 33.8 | 33.0 |
|  | 80K < 100k |  | 9.8 | 10.1 | 11.6 | 11.3 |
|  | 50k < 80k |  | 16.8 | 17.4 | 20.5 | 20.5 |
|  | 20k < 50k |  | 23.3 | 22.1 | 25.9 | 26.5 |
|  | Less than 20k |  | 8 | 7.0 | 8.3 | 8.7 |
|  | Missing |  | 14.8 | 14.8 | 0.0 | 0.0 |
| Educational attainment | Tertiary^a^ |  | 22.4 | 23.3 | 23.3 | 24.3 |
|  | Vocational^b^ |  | 45.5 | 45.7 | 47.3 | 47.6 |
|  | Student^c^ |  | 7.3 | 7.7 | 6.2 | 5.5 |
|  | Secondary^d^ |  | 24.8 | 23.3 | 23.2 | 22.7 |
| Inadequate dentition | No |  | 77.2 | 81.0 | 89.0 | 89.0 |
|  | Yes |  | 9.6 | 10.1 | 11.0 | 11.0 |
|  | Missing/ Edentate^$^ |  | 13.2 | 9.0 (Missing) | 0.0 | 0.0 |
| Self-rated oral health | Excellent/Very Good/Good |  | 75.9 | 79.6 | 79.5 | 79.2 |
|  | Poor/Very Poor |  | 19.3 | 20.2 | 20.5 | 20.8 |
|  | Missing |  | 4.8 | 0.1 |  |  |
| Remoteness | Major city |  | 70.5 | 71.0 | 70.8 | 70.5 |
|  | Inner regional |  | 18.7 | 18.4 | 18.7 | 18.8 |
|  | Outer regional |  | 8.4 | 8.2 | 8.3 | 8.5 |
|  | Remote/Very Remote |  | 2.0 | 2.0 | 2.2 | 2.2 |
|  | Missing |  | 0.5 | 0.5 |  |  |
| LGA Allocation | Yes |  | 96.2 | 96.0 | 100 | 100 |
|  | No |  | 3.8 | 4.0 | 0 | 0 |
| Weighted Percentages; a: Bachelor/honors degree or more; b: Advanced diploma, diploma, associate degree, certificate level, and other qualifications; c: None completed but studying at university, TAFE apprentice, secondary school; d: No post-secondary qualification & not currently studying | | | | | | |
