# Supplementary material for: Area-level income inequality and oral health among Australian adults—A population-based multilevel study
Source: PLoS One. 2018 Jan 24;13(1):e0191438. doi: 10.1371/journal.pone.0191438 (PMC5783384; doi:10.1371/journal.pone.0191438)
Supplement: S2 Table — (DOCX) [file pone.0191438.s005.docx]

S2. Table: Multilevel logistic regression analysis for the association between LGA level income inequality and inadequate dentition (No. of Areas= 428; N of individuals=4,768)

|  |  | Null Model | | Model 1 | | Model 2 | | Model 3 | | Model 4 | | Model 5 |  |  |
| --- | --- | --- | --- | --- | --- | --- | --- | --- | --- | --- | --- | --- | --- | --- |
|  | Categories | OR | 95% CI | OR | 95% CI | OR | 95% CI | OR | 95% CI | OR | 95% CI | OR | 95%CI |  |
| Income Inequality (Gini) | Low |  |  | 1 |  | 1 |  | 1 |  | 1 |  | 1 |  |  |
|  | Medium |  |  | 1.10 | 0.89, 1.37 | 0.87 | 0.69, 1.09 | 0.87 | 0.69, 1.08 | 0.86 | 0.69, 1.09 | 0.88 | 0.70, 1.11 |  |
|  | High |  |  | 0.59 | 0.46, 0.75 | 0.43 | 0.33, 0.56 | 0.58 | 0.43, 0.77 | 0.60 | 0.45, 0.81 | 0.64 | 0.48, 0.87 |  |
| Mean weekly household income | High |  |  |  |  |  |  | 1 |  | 1 |  | 1 |  |  |
|  | Medium |  |  |  |  |  |  | 1.59 | 1.24, 2.05 | 1.43 | 1.11, 1.85 | 1.44 | 1.12, 1.86 |  |
|  | Low |  |  |  |  |  |  | 1.73 | 1.30, 2.31 | 1.40 | 1.04, 1.87 | 1.37 | 1.00, 1.88 |  |
| Age | 1-year change |  |  |  |  | 1.09 | 1.08, 1.10 | 1.09 | 1.08, 1.10 | 1.07 | 1.06, 1.08 | 1.07 | 1.06, 1.08 |  |
| Sex | Male |  |  |  |  | 1 |  | 1 |  | 1 |  | 1 |  |  |
|  | Female |  |  |  |  | 0.91 | 0.75, 1.09 | 0.91 | 0.76, 1.10 | 0.79 | 0.65, 0.96 | 0.79 | 0.65, 0.96 |  |
| Household Income | $100K and above |  |  |  |  |  |  |  |  | 1 |  | 1 |  |  |
|  | 80K < 100k |  |  |  |  |  |  |  |  | 1.79 | 1.12, 2.86 | 1.79 | 1.13, 2.87 |  |
|  | 50k < 80k |  |  |  |  |  |  |  |  | 2.55 | 1.75, 3.71 | 2.56 | 1.76, 3.73 |  |
|  | 20k < 50k |  |  |  |  |  |  |  |  | 3.91 | 2.75, 5.58 | 3.97 | 2.78, 5.66 |  |
|  | Less than 20k |  |  |  |  |  |  |  |  | 6.49 | 4.38, 9.61 | 6.56 | 4.42, 9.72 |  |
| Remoteness | Major City |  |  |  |  |  |  |  |  |  |  | 1 |  |  |
|  | Inner Regional |  |  |  |  |  |  |  |  |  |  | 1.10 | 0.84, 1.43 |  |
|  | Outer Regional |  |  |  |  |  |  |  |  |  |  | 1.04 | 0.75, 1.44 |  |
|  | Remote/Very Remote |  |  |  |  |  |  |  |  |  |  | 1.55 | 0.98, 2.44 |  |

Model 1: Unadjusted; Model 2: Adjusted for age and sex; Model 3: Adjusted for age, sex, LGA level mean income; Model 4: Adjusted for age, sex, LGA level mean income and household income; ICC: Intra-class Coefficient, MOR: Median Odds Ratio, DIC: Deviance Information Criterion
