# Supplementary material for: Area-level income inequality and oral health among Australian adults—A population-based multilevel study
Source: PLoS One. 2018 Jan 24;13(1):e0191438. doi: 10.1371/journal.pone.0191438 (PMC5783384; doi:10.1371/journal.pone.0191438)
Supplement: S3 Table — (DOCX) [file pone.0191438.s006.docx]

S3. Table. Multilevel logistic regression analysis for the association between LGA level income inequality and poor self-rated oral health (N of Areas =435; N of individuals=5,165)

|  |  | Null Model | | Model 1 | | Model 2 | | Model 3 | | Model 4 | | Model 5 | |
| --- | --- | --- | --- | --- | --- | --- | --- | --- | --- | --- | --- | --- | --- |
|  | Categories | OR | 95% CI | OR | 95% CI | OR | 95% CI | OR | 95% CI | OR | 95% CI | OR | 95% CI |
| Income Inequality (Gini) | Low |  |  | 1 |  | 1 |  | 1 |  | 1 |  | 1 |  |
|  | Medium |  |  | 0.93 | 0.79, 1.10 | 0.92 | 0.78, 1.09 | 0.93 | 0.78, 1.10 | 0.92 | 0.77, 1.10 | 0.95 | 0.80, 1.13 |
|  | High |  |  | 0.77 | 0.65, 0.91 | 0.76 | 0.64, 0.90 | 0.89 | 0.73, 1.08 | 0.90 | 0.73, 1.10 | 0.92 | 0.74, 1.14 |
| Mean weekly household income | High |  |  |  |  |  |  | 1 |  | 1 |  | 1 |  |
|  | Medium |  |  |  |  |  |  | 1.20 | 1.01, 1.44 | 1.06 | 0.88, 1.28 | 1.10 | 0.91, 1.33 |
|  | Low |  |  |  |  |  |  | 1.48 | 1.20, 1.83 | 1.19 | 0.95, 1.49 | 1.29 | 1.02, 1.65 |
| Age | 1-year change |  |  |  |  | 1.01 | 1.00, 1.01 | 1.00 | 1.00, 1.01 | 1.00 | 0.99, 1.00 | 1.00 | 0.99, 1.00 |
| Sex | Male |  |  |  |  | 1 |  | 1 |  | 1 |  | 1 |  |
|  | Female |  |  |  |  | 0.86 | 0.74, 0.98 | 0.86 | 0.75, 0.99 | 0.79 | 0.68, 0.91 | 0.79 | 0.68, 0.91 |
| Household Income | $100K and above |  |  |  |  |  |  |  |  | 1 |  | 1 |  |
|  | 80K < 100k |  |  |  |  |  |  |  |  | 1.39 | 1.06, 1.81 | 1.39 | 1.07, 1.81 |
|  | 50k < 80k |  |  |  |  |  |  |  |  | 1.78 | 1.43, 2.20 | 1.78 | 1.44, 2.21 |
|  | 20k < 50k |  |  |  |  |  |  |  |  | 2.59 | 2.10, 3.19 | 2.62 | 2.12, 3.22 |
|  | Less than 20k |  |  |  |  |  |  |  |  | 4.06 | 3.12, 5.29 | 4.08 | 3.13, 5.31 |
| Remoteness | Major City |  |  |  |  |  |  |  |  |  |  | 1 |  |
|  | Inner Regional |  |  |  |  |  |  |  |  |  |  | 0.87 | 0.71, 1.06 |
|  | Outer Regional |  |  |  |  |  |  |  |  |  |  | 0.94 | 0.74, 1.19 |
|  | Remote/Very Remote |  |  |  |  |  |  |  |  |  |  | 1.46 | 1.06, 2.01 |

Model 1: Unadjusted; Model 2: Adjusted for age and sex; Model 3: Adjusted for age, sex, LGA level mean income; Model 4: Adjusted for age, sex, LGA level mean income and household income; ICC: Intra-class Coefficient, MOR: Median Odds Ratio, DIC: Deviance Information Criterion
