# Supplementary material for: Area-level income inequality and oral health among Australian adults—A population-based multilevel study
Source: PLoS One. 2018 Jan 24;13(1):e0191438. doi: 10.1371/journal.pone.0191438 (PMC5783384; doi:10.1371/journal.pone.0191438)
Supplement: S4 Table — (DOCX) [file pone.0191438.s007.docx]

S4. Table. Sensitivity analysis (sensitivity analysis -1) to investigate differences in the associations of income inequality and oral health outcomes after excluding:

| Inadequate Dentition | | | | | | | | | | | |
| --- | --- | --- | --- | --- | --- | --- | --- | --- | --- | --- | --- |
|  |  |  | Model 1 | | Model 2 | | Model 3 | | Model 4 | |  |
|  |  |  | OR | 95% CI | OR | 95% CI | OR | 95% CI | OR | 95% CI |  |
| Full cases | Income Inequality | Low | 1 |  | 1 |  | 1 |  | 1 |  |  |
|  |  | Medium | 1.10 | 0.89, 1.37 | 0.87 | 0.69, 1.09 | 0.87 | 0.69, 1.08 | 0.86 | 0.69, 1.09 |  |
|  |  | High | 0.59 | 0.46, 0.75 | 0.43 | 0.33, 0.56 | 0.58 | 0.43, 0.77 | 0.60 | 0.45, 0.81 |  |
| Excluding singletons |  | Low | 1 |  | 1 |  | 1 |  | 1 |  |  |
|  |  | Medium | 1.08 | 0.87, 1.35 | 0.85 | 0.68, 1.07 | 0.85 | 0.68, 1.07 | 0.85 | 0.68, 1.07 |  |
|  |  | High | 0.58 | 0.45, 0.75 | 0.43 | 0.33, 0.56 | 0.58 | 0.44, 0.78 | 0.61 | 0.46, 0.82 |  |
| Minimum 5 per LGA |  | Low | 1 |  | 1 |  | 1 |  | 1 |  |  |
|  |  | Medium | 1.08 | 0.87, 1.35 | 0.84 | 0.67, 1.06 | 0.84 | 0.67, 1.05 | 0.85 | 0.67, 1.07 |  |
|  |  | High | 0.58 | 0.45, 0.74 | 0.42 | 0.32, 0.55 | 0.57 | 0.42, 0.76 | 0.60 | 0.45, 0.82 |  |
|  |  |  |  |  |  |  |  |  |  |  |  |
| Poor Self-rated oral health | | | | | | | | | | | |
| Full cases | Income Inequality | Low | 1 |  | 1 |  | 1 |  | 1 |  |  |
|  |  | Medium | 0.93 | 0.79, 1.10 | 0.92 | 0.78, 1.09 | 0.93 | 0.78, 1.10 | 0.92 | 0.77, 1.10 |  |
|  |  | High | 0.77 | 0.65, 0.91 | 0.76 | 0.64, 0.90 | 0.89 | 0.73, 1.08 | 0.90 | 0.73, 1.10 |  |
| Excluding singletons |  | Low | 1 |  | 1 |  | 1 |  | 1 |  |  |
|  |  | Medium | 0.92 | 0.78, 1.09 | 0.91 | 0.77, 1.08 | 0.92 | 0.77, 1.09 | 0.92 | 0.77, 1.09 |  |
|  |  | High | 0.77 | 0.65, 0.91 | 0.76 | 0.64, 0.91 | 0.90 | 0.74, 1.10 | 0.91 | 0.74, 1.12 |  |
| Minimum 5 per LGA |  | Low | 1 |  | 1 |  | 1 |  | 1 |  |  |
|  |  | Medium | 0.94 | 0.79, 1.11 | 0.92 | 0.78, 1.10 | 0.93 | 0.78, 1.10 | 0.93 | 0.77, 1.11 |  |
|  |  | High | 0.77 | 0.65, 0.92 | 0.76 | 0.64, 0.91 | 0.89 | 0.72, 1.09 | 0.90 | 0.72, 1.11 |  |

1. Singletons for inadequate dentition (13.08%) and poor self-rated oral health (14.3%). Remaining sample: Inadequate dentition (n=4,712; LGAs= 372) Poor self-rated oral health (n=5,103; LGAs= 373).
2. Applying a minimum cutoff of 5 individuals per LGA: Remaining sample: Inadequate dentition (n=4,510; LGAs= 280) Poor self-rated oral health (n=4,882; LGAs= 280)

Model 1: Unadjusted; Model 2: Adjusted for age and sex; Model 3: Adjusted for age, sex, LGA level mean income; Model 4: Adjusted for age, sex, LGA level mean income and household income
