# Supplementary material for: Area-level income inequality and oral health among Australian adults—A population-based multilevel study
Source: PLoS One. 2018 Jan 24;13(1):e0191438. doi: 10.1371/journal.pone.0191438 (PMC5783384; doi:10.1371/journal.pone.0191438)
Supplement: S6 Table — (DOCX) [file pone.0191438.s009.docx]

S6. Table. Sensitivity analysis (sensitivity analysis-3) to investigate variations in the associations between area-level income inequality and inadequate dentition and poor self-rated oral health after adjusting for educational attainment, tertiles of IRSAD scores and area-remoteness, results from multilevel multivariable logistic regression models

| Inadequate dentition (n: 4,768; LGAs 428) | | | | | | | | | | | | | | |
| --- | --- | --- | --- | --- | --- | --- | --- | --- | --- | --- | --- | --- | --- | --- |
|  |  | Null Model | | Model 1 | | Model 2 | | Model 3 | | Model 4 | | Model 5 | | |
|  | Categories | OR | 95% CI | OR | 95% CI | OR | 95% CI | OR | 95% CI | OR | 95% CI | OR | 95% CI |  |
| Income Inequality (Gini) | Low |  |  | 1 |  | 1 |  | 1 |  | 1 |  | 1 |  |  |
|  | Medium |  |  | 1.10 | 0.89, 1.37 | 0.87 | 0.69, 1.09 | 0.87 | 0.69, 1.08 | 0.86 | 0.69, 1.09 | 0.90 | 0.72, 1.13 |  |
|  | High |  |  | 0.59 | 0.46, 0.75 | 0.43 | 0.33, 0.56 | 0.58 | 0.43, 0.77 | 0.60 | 0.45, 0.81 | 0.64 | 0.47, 0.86 |  |
| Poor self-rated oral health (n: 5,165; LGAs: 435) | | | | | | | | | | | | | | |
|  |  | OR | 95% CI | OR | 95% CI | OR | 95% CI | OR | 95% CI | OR | 95% CI | OR | 95% CI |  |
| Income Inequality (Gini) | Low |  |  | 1 |  | 1 |  | 1 |  | 1 |  | 1 |  |  |
|  | Medium |  |  | 0.93 | 0.79, 1.10 | 0.92 | 0.78, 1.09 | 0.93 | 0.78, 1.10 | 0.92 | 0.77, 1.10 | 0.94 | 0.78, 1.12 |  |
|  | High |  |  | 0.77 | 0.65, 0.91 | 0.76 | 0.64, 0.90 | 0.89 | 0.73, 1.08 | 0.90 | 0.73, 1.10 | 0.95 | 0.76, 1.19 |  |

Model 1: Unadjusted; Model 2: Adjusted for age and sex; Model 3: Adjusted for age, sex, LGA level mean income; Model 4: Adjusted for age, sex, LGA level mean income and household income; Model 4: Adjusted for age, sex, LGA level mean income and household income; Model 5: Adjusted for education, remoteness, and Index of Relative Socio-economic Advantage and Disadvantage (IRSAD)
