# Supplementary material for: Area-level income inequality and oral health among Australian adults—A population-based multilevel study
Source: PLoS One. 2018 Jan 24;13(1):e0191438. doi: 10.1371/journal.pone.0191438 (PMC5783384; doi:10.1371/journal.pone.0191438)
Supplement: S7 Table — (DOCX) [file pone.0191438.s010.docx]

S7. Table. Sensitivity analysis (sensitivity analysis-4) to investigate for variation in the association between LGA-level income inequality and inadequate dentition according to a categorization of LGA-level income inequality derived through k-cluster analysis.

| Income inequality | Odds Ratio (95% CI) for inadequate dentition compared to no inadequate dentition |
| --- | --- |
| Low (0.292 – 0.370) | 1 |
| Medium (0.371 – 0.402) | 0.85 (0.69, 1.05) |
| High (0.403 – 0.474) | 0.54 (0.36, 0.81) |

Adjusted for age, sex, LGA-level equivalised weekly mean household income and household income
