# Supplementary material for: Area-level income inequality and oral health among Australian adults—A population-based multilevel study
Source: PLoS One. 2018 Jan 24;13(1):e0191438. doi: 10.1371/journal.pone.0191438 (PMC5783384; doi:10.1371/journal.pone.0191438)
Supplement: S8 Table — (DOCX) [file pone.0191438.s011.docx]

S8. Table. Sensitivity analysis (sensitivity analysis-5) to investigate for residual confounding by LGA-level mean household income and household income in the association between LGA-level income inequality and inadequate dentition. Values of LGA-level mean household income and 16 categories of household income were included on continuous scale

| Income inequality | Odds Ratio (95% CI) for inadequate dentition compared to no inadequate dentition |
| --- | --- |
| Low | 1 |
| Medium | 0.91 (0.72, 1.14) |
| High | 0.68 (0.50, 0.92) |

Adjusted for age, sex, LGA-level equivalised weekly mean household income, household income and geographic remoteness.
